# Characterization of SARS‐CoV‐2 humoral immune response in a subject with unique sampling: A case report

**DOI:** 10.1002/iid3.910

**Published:** 2023-06-14

**Authors:** Melanie R. Walker, Manja Idorn, Anja Bennett, Max Søgaard, Ali Salanti, Sisse B. Ditlev, Lea Barfod

**Affiliations:** ^1^ Department of Immunology and Microbiology, Centre for Medical Parasitology, Faculty of Health and Medical Sciences University of Copenhagen Copenhagen Denmark; ^2^ Department of Biomedicine Aarhus University Aarhus Denmark; ^3^ Department of Mammalian Expression Global Research Technologies Måløv Denmark; ^4^ Expres2ion Biotechnologies Hørsholm Denmark; ^5^ Copenhagen Center for Translational Research Bispebjerg Hospital Copenhagen Denmark

**Keywords:** antibody, antibody isotypes, antibody subclasses, case report, COVID‐19, humoral immunity, IgG, SARS‐CoV‐2

## Abstract

**Background:**

The development of vaccine candidates for COVID‐19, and the administration of booster vaccines, has meant a significant reduction in COVID‐19 related deaths world‐wide and the easing of global restrictions. However, new variants of SARS‐CoV‐2 have emerged with less susceptibility to vaccine induced immunity leading to breakthrough infections among vaccinated people. It is generally acknowledged that immunoglobulins play the major role in immune‐protection, primarily through binding to the SARS‐COV‐2 receptor binding domain (RBD) and thereby inhibiting viral binding to the ACE2 receptor. However, there are limited investigations of anti‐RBD isotypes (IgM, IgG, IgA) and IgG subclasses (IgG1–4) over the course of vaccination and breakthrough infection.

**Method:**

In this study, SARS‐CoV‐2 humoral immunity is examined in a single subject with unique longitudinal sampling. Over a two year period, the subject received three doses of vaccine, had two active breakthrough infections and 22 blood samples collected. Serological testing included anti‐nucleocapsid total antibodies, anti‐RBD total antibodies, IgG, IgA, IgM and IgG subclasses, neutralization and ACE2 inhibition against the wildtype (WT), Delta and Omicron variants.

**Results:**

Vaccination and breakthrough infections induced IgG, specifically IgG1 and IgG4 as well as IgM and IgA. IgG1 and IgG4 responses were cross reactive and associated with broad inhibition.

**Conclusion:**

The findings here provide novel insights into humoral immune response characteristics associated with SARS‐CoV‐2 breakthrough infections.

## Graphical abstract

In a Danish subject with unique longitudinal sampling, SARS‐CoV‐2 humoral immunity over a 2‐year period throughout three doses of vaccine and two breakthrough infections is examined. A total of 22 samples are tested for antinucleocapsid total antibodies, anti‐RBD total antibodies, IgG, IgA, IgM, and IgG subclasses, neutralization and ACE2 inhibition against the wildtype (WT), Delta and Omicron variants. We find that vaccination and breakthrough infection induce cross‐reactive inhibitory IgG1 and IgG4 antibodies, highlighting the need to characterize these responses, as future vaccine design efforts may seek to promote such anti‐RBD IgG1 and IgG4 responses.

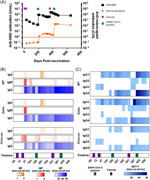

## 1 NOTES AND INSIGHTS

The development of vaccine candidates for COVID‐19, and the administration of booster vaccines, has meant a significant reduction in COVID‐19 related deaths world‐wide and the easing of global restrictions. However, new variants of SARS‐CoV‐2 have emerged with less susceptibility to vaccine induced immunity leading to breakthrough infections among vaccinated people.^1^ It is generally acknowledged that immunoglobulins (Ig) play the major role in immune‐protection, primarily through binding to the SARS‐COV‐2 receptor binding domain (RBD) and thereby inhibiting viral binding to the ACE2 receptor.^2^ However, there are limited investigations of anti‐RBD isotypes (IgM, IgG, IgA) and IgG subclasses (IgG1–4) over the course of vaccination and breakthrough infection.^2^, ^3^, ^4^, ^5^, ^6^ Studying these associations at the individual level is important for understanding immune responses induced by current vaccines and for the future development of antibody‐based vaccines, as varied antibody subclasses and isotypes have been found to be important in controlling different pathogenic infections.^2^, ^7^, ^8^, ^9^

In this study, SARS‐CoV‐2 humoral immunity is examined longitudinally in a 47‐year‐old immunocompetent woman who was previously found to have broad SARS‐CoV‐2 RBD‐specific IgG and RBD/ACE2 inhibition 27 days after two doses of vaccine (Supporting Information: Figure S1).^10^ Over a 2‐year period, this individual had 22 samples collected, three doses of Pfizer‐BioNTech BNT162b2, and two documented episodes of SARS‐CoV‐2 breakthrough infection (Supporting Information: Figure S1, Table S1). During the time of the first infection, the Delta variant account for 92%–98% of infections in Denmark, and during the second infection, BA.2 accounted for 96%–98% of infections in Denmark (Supporting Information: Table S1).

**Figure 1:**
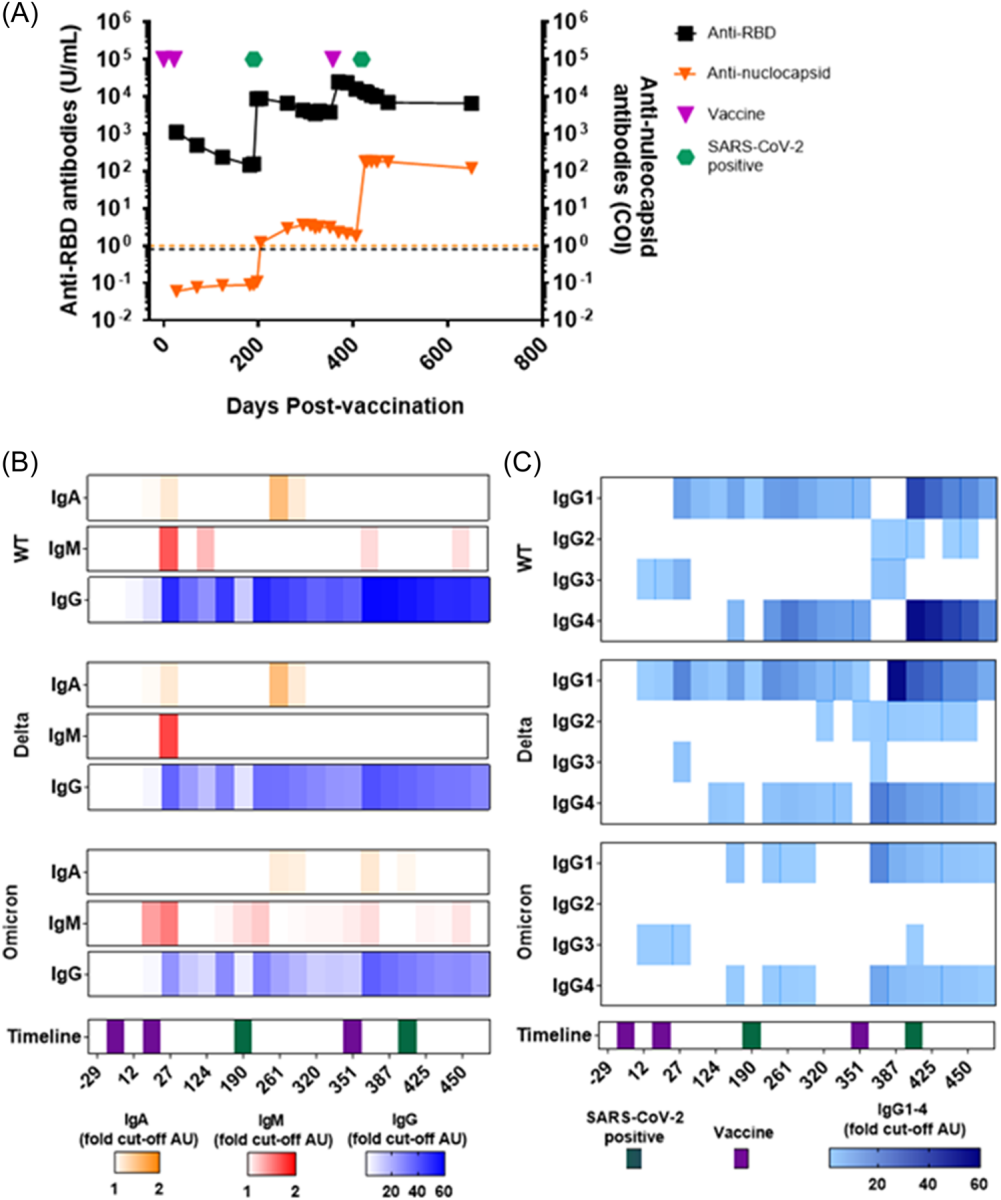
Total (IgA + IgG+ IgM) antibody, isotypes and subclass levels after vaccination and infection. RBD (black) and nucleocapsid (orange) ‐specific antibody levels were measured longitudinally after vaccination (purple triangle) and infection (green hexagon). (A) Heat maps representing longitudinal patterns of anti‐RBD antibody isotype. (B) and subclass. (C) utilization (AU) against three SARS‐CoV‐2 variants. Anti‐RBD IgA is represented in orange, anti‐RBD IgM in red and anti‐RBD IgG and IgG1–4 (signal/noise) in blue. Lower magnitudes are lighter and higher magnitudes are darker (see key). A timeline of events (infections, green; vaccinations, purple) is also shown. Days postvaccination are indicated below each heat map. All results were generated from duplicate measures from serum samples collected.

We first used Elecsys assays (Roche Diagnostics) to assess RBD‐specific and nucleocapsid‐specific total serum (IgA + IgG + IgM) antibodies longitudinally in subject KSID to determine serological responses after infection and vaccination (Figure 1A).

The antinucleocapsid response, a useful tool to distinguish between serological responses to infection and vaccination response, was not detected until the first infection indicating no previous SARS‐CoV‐2 infection (Figure 1A). Once induced, responses remained relatively stable for 200 days. Upon the second infection, antibody levels increased 90 fold and were sustained for another 225 days.

After two doses of Pfizer‐BioNTech BNT162b2, anti‐WT RBD antibodies were induced (Figure 1A) but started to decline at the next sampling time point. By 7 days after the first reported infection, RBD‐specific antibodies had increased rapidly, more than eight times higher than the vaccination peak. Nevertheless, this second boost was not sustained and started to decline 71 days after the first reported infection (261 days postvaccination). Upon administration of the third booster, RBD‐specific antibody responses increased 2.7 fold higher than the last peak from the first infection, but again started to decrease upon the next sampling time point. Upon second infection, no increase in RBD specific antibody responses was observed. Nevertheless, anti‐RBD antibodies remained relatively high and sustained up to 650 days postvaccination.

Next, RBD‐specific IgM, IgG, and IgA responses were examined against WT, Delta, and Omicron variants to determine which isotype responses were being generated after vaccination and infection (Figure 1B).

IgM and IgA were detectable against all variants at Day 27 after the second dose of vaccine and were again present after each infection and vaccination. Nevertheless, throughout the collection period, both IgM and IgA responses were low and transient. After the third dose of vaccine, IgA was observed against Omicron only, and no IgA was observed to any variant after the second infection.

Anti‐RBD IgG was observed against all variants longitudinally but was 1.3–3.3 times higher against the WT than the Delta and Omicron variants throughout the entire collection period (Supporting Information: Table S2). Nevertheless, IgG responses followed the same pattern against all variants with boosting followed by decline after each vaccination and/or infection as observed for total antibody response using the Elecsys assay above.

Next, IgG subclass utilization was examined to understand whether specific subclasses are induced preferentially (Figure 1C). IgG3 responses were the first to be observed, appearing 12 days after the first vaccination. However, IgG3 responses were transient, and declined after the second dose of vaccine. IgG3 was then boosted after the third vaccine dose, and for the Omicron variant, after the second infection, however these responses quickly declined. The initial IgG3 responses were followed by IgG1 which had a similar pattern of induction as total IgG where each vaccination and/or infection caused a boost in IgG levels which then declined. Of note is the response towards the Omicron variant which increased after the third dose of vaccine but not after the second infection (likely an Omicron BA.2 infection). Limited anti‐RBD IgG2 was detected throughout infection, and the responses that were detected were only marginally above cut‐off. Interestingly, there was a strong IgG4 response throughout the collection period. This response first appeared at 180 days after the first vaccination, and before the first infection. IgG4 levels increased 3 fold after the first infection and then peaked after the third vaccination however, no boosting was observed after the second infection.

Together we show a strong IgG response, particularly IgG1 and IgG4 is associated with vaccination and infection.

**Figure 2:**
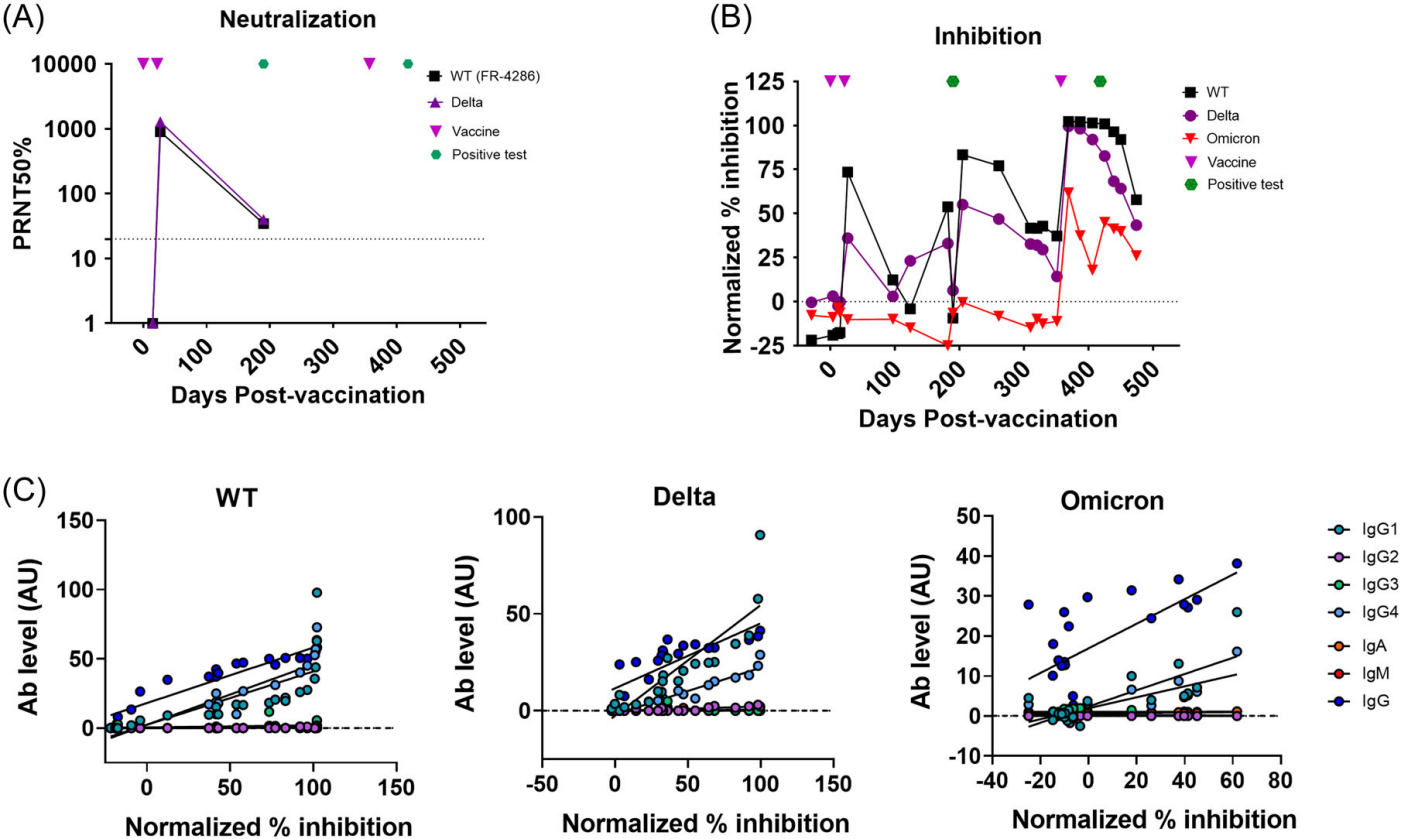
Inhibition of serum against SARS‐CoV‐2 RBD variants. Inhibition activity of KSID serum against the WT and Delta was measured using a neutralization assay. (A) and against WT, Delta and Omicron variants using an ACE2 inhibition assay. (B) The relationship between ACE2 inhibition and isotype and subclass levels (AU) specific for each variant, were measured by linear regression (black line), *R* ^2^ and *p* values for the linear regression are reported in Supporting Information: Table S4. RBD, receptor binding domain; WT, wildtype.

To assess the variant transcending capacity of the serum, neutralization assays were performed on the WT and Delta variant at 15, 27, and 190 days postfirst vaccination (Figure 2A). Neutralization against both the WT and Delta variant was first observed at 27 days and again at 190 days. However, this response at 190 days had declined when compared to the 27 day time point by 25 and 32 fold for the WT and Delta variant, respectively.

To further investigate the cross‐reactive and inhibitory nature of the humoral immune response, RBD‐specific ACE2 competition assays were performed against the WT, Delta, and Omicron variants over the complete time course (Figure 2B). Inhibition responses followed those of binding where responses increased and waned after each vaccination or infection. In general, inhibition against the WT was on average 1.35 fold and 2.7 fold higher than that of the Delta and Omicron variants respectively (Supporting Information: Table S3). However, upon third vaccination, inhibition against the Delta variant increased to WT levels. Interestingly, Omicron inhibition was only detected after the third vaccine dose. Furthermore, a boost in inhibition toward Omicron, but not WT and Delta, was observed after the second SARS‐CoV‐2 infection at 425 days which is likely due to an infection with Omicron BA.2 variant, which was circulating at the time.

Next, the relationship between inhibition and antibody subtype and isotype responses were examined (Figure 2C and Supporting Information: Table S4). For all variants, inhibition correlated significantly with IgG, specifically IgG1 and IgG4 indicating a protective IgG1 and IgG4 response.

Many SARS‐CoV‐2 studies have focused on the induction and decay of IgG responses only.^2^, ^10^ In this study we measured RBD‐specific IgM, IgA and IgG, and IgG subclasses against WT, Delta, and Omicron variants. We found that IgM and IgA responses were low, transient and did not correlate with inhibition. Nevertheless, these responses were cross‐reactive and present after breakthrough infection and booster dose. This is an important observation as early IgM and IgA responses may be protective. IgM has been shown to play an important role in the maturation of B‐cell responses regulating B‐cell tolerance as well as class switching to IgG and IgA^7^ and IgA toward SARS‐CoV‐2 has been observed after breakthrough infections indicating a protective response.^4^, ^5^

In terms of IgG subclasses, IgG3 appeared first in the course of infection, shortly after first vaccination. However, this response was transient and was followed immediately by an IgG1 response which in turn was followed by IgG2 and IgG4. Several SARS‐CoV‐2 studies have observed similar trends of short and transient IgG3 responses after vaccination or infection and these results support the hypothesis of proximal IgG3 and distal IgG4.^3^, ^8^, ^9^ IgG1 has been implicated as the major mediator of protection in many viral infections.^8^, ^9^ In viral infection, IgG4 induction is infrequent^8^, ^9^ and is often formed following long term or repeated exposures but has been observed against SARS‐CoV‐2 RBD 5–7 months after repeated SARS‐CoV‐2 mRNA vaccination.^3^

RBD‐specific IgG binding, specifically IgG1 and IgG4 correlated with RBD/ACE2 inhibition for WT, Delta, and Omicron variants. IgG4 has previously been found in subjects with high inhibition after third vaccination but was associated with reduced antibody effector functions.^3^ These responses were detectable over the entire sampling period for the WT and Delta variants but not until the third booster, for Omicron after which responses were lower than those of the Delta and WT, as observed previously.^10^

This study provides important insight into the acquisition and maintenance of antibody isotypes and subclasses after vaccination and breakthrough infection and highlights the need to characterize these responses for future vaccine design efforts.

## AUTHORS CONTRIBUTIONS

**Melanie R. Walker**: Conceptualization; methodology; formal analysis; investigation; data curation; writing—original draft preparation; writing—review and editing. **Lea Barfod**: Conceptualization; methodology; formal analysis; investigation; resources; funding acquisition. **Sisse B. Ditlev**: Conceptualization; methodology; formal analysis; investigation; resources; funding acquisition; writing—review and editing. **Manja Idorn**: Methodology; formal analysis; resources; data curation. **Anja Bennett**: Methodology; resources. **Max Søgaard**: Methodology; resources. **Ali Salanti**: Methodology; resources; funding acquisition. All authors have read and agreed to the published version of the manuscript.

## CONFLICT OF INTEREST STATEMENT

The authors declare no conflict of interest.

## ETHICS STATEMENT

Human research ethics approvals were obtained for all samples from the Regional Research Ethics Committees for the Capital Region of Denmark (Protocols H‐4‐2013‐083, H‐20035553 and H‐20034367) and all patients gave informed consent. All methods were performed in accordance with the relevant guidelines and regulations and in accordance with the Declaration of Helsinki. Written consent for case publication was obtained from the patient.

## Supporting information

Supporting information.

## Data Availability

All data generated or analyzed during this study are included in this published article (and its Supporting Information files).

## References

[1] Lipsitch M , Krammer F , Regev‐Yochay G , Lustig Y , Balicer RD . SARS‐CoV‐2 breakthrough infections in vaccinated individuals: measurement, causes and impact. Nat Rev Immunol. 2022;22(1):57‐65. 34876702 10.1038/s41577-021-00662-4 PMC8649989

[2] Huang AT , Garcia‐Carreras B , Hitchings MDT , et al. A systematic review of antibody mediated immunity to coronaviruses: kinetics, correlates of protection, and association with severity. Nat Commun. 2020;11(1):4704. 32943637 10.1038/s41467-020-18450-4 PMC7499300

[3] Irrgang P , Gerling J , Kocher K , et al. Class switch toward noninflammatory, spike‐specific IgG4 antibodies after repeated SARS‐CoV‐2 mRNA vaccination. Sci Immunol. 2023;8(27):eade2798. 36548397 10.1126/sciimmunol.ade2798 PMC9847566

[4] Liu H , Varvel S , Chen G , et al. Simultaneous measurement of multiple variant‐specific SARS‐CoV‐2 neutralizing antibodies with a multiplexed flow cytometric assay. Front Immunol. 2022;13:1039163. 36505453 10.3389/fimmu.2022.1039163 PMC9732243

[5] Hennings V , Thörn K , Albinsson S , et al. The presence of serum anti‐SARS‐CoV‐2 IgA appears to protect primary health care workers from COVID‐19. Eur J Immunol. 2022;52(5):800‐809. 35128644 10.1002/eji.202149655 PMC9087394

[6] Chen W , Zhang L , Li J , et al. The kinetics of IgG subclasses and contributions to neutralizing activity against SARS‐CoV‐2 wild‐type strain and variants in healthy adults immunized with inactivated vaccine. Immunology. 2022;167(2):221‐232. 35751471 10.1111/imm.13531 PMC9349727

[7] Kubagawa H , Honjo K , Ohkura N , et al. Functional roles of the IgM Fc receptor in the immune system. Front Immunol. 2019;10:945. 31130948 10.3389/fimmu.2019.00945 PMC6509151

[8] Walker MR , Eltahla AA , Mina MM , Li H , Lloyd AR , Bull RA . Envelope‐specific IgG3 and IgG1 responses are associated with clearance of acute hepatitis C virus infection. Viruses. 2020;12(1):75. 31936235 10.3390/v12010075 PMC7019651

[9] Vidarsson G , Dekkers G , Rispens T . IgG subclasses and allotypes: from structure to effector functions. Front Immunol. 2014;5:520. 25368619 10.3389/fimmu.2014.00520 PMC4202688

[10] Walker MR , Podlekareva D , Johnsen S , et al. SARS‐CoV‐2 RBD‐specific antibodies induced early in the pandemic by natural infection and vaccination display cross‐variant binding and inhibition. Viruses. 2022;14(9):1861. 36146667 10.3390/v14091861 PMC9503696

