# Supplementary material for: Characterization of SARS‐CoV‐2 humoral immune response in a subject with unique sampling: A case report
Source: Immun Inflamm Dis. 2023 Jun 14;11(6):e910. doi: 10.1002/iid3.910 (PMC10266136; doi:10.1002/iid3.910)
Supplement: Supplementary file 1 — Supporting information. [file IID3-11-e910-s001.docx]

**Supplementary information**

# Materials and Methods

# Subject and sample characteristics

Patient KSID was a 47 year-old immunocompetent woman. KSID was recruited from Bispebjerg Hospital, Copenhagen, Denmark as part of 21 healthcare workers vaccinated with two doses of Pfizer-BioNTech BNT162b2 (Comirnaty®) during the screening of SARS-CoV-2 antibodies in hospital staff employed in the Capital Region of Denmark (1). She received her first dose of BNT162b2 on the 13^th^ of January 2021 and the second dose on the 4^th^ of February 2021 (Table 1). On the 21st of July 2021, she developed a headache and loss of taste and smell. The next day (22^nd^ of July 2021) she tested positive via PCR for SARS-CoV-2. All symptoms resolved after 10 days. She received her third dose of BNT162b2 on the 5^th^ of January 2022. On the 7^th^ of March 2022 she developed a headache and tested positive for SARS-CoV-2 via PCR again. Within a week she had fully recovered.

Blood samples were collected before vaccination (-29) and longitudinally at 22 time points from January 2021 to October 2022 (Table 1). Blood samples were collected in serum vacutainers and separated by centrifugation. Serum was then collected and stored at -80°C until use. Questionnaires regarding symptoms of infection, type of work and demographics were completed and entered into a database as previously described (1, 2).

**Table S1. Longitudinal sampling of subject KSID**

| **Date** | **Time point (Days post first vaccination)** | **Sampling** | **Event** | **Dominant variant** (3, 4) |
| --- | --- | --- | --- | --- |
| 15.12.2020 | -29 | Y |  | WT |
| 13.01.2021 | 0 | N | Vaccine (Pfizer-BioNTech BNT162b2) |  |
| 17.01.2021 | 4 | Y |  |  |
| 25.01.2021 | 12 | Y |  |  |
| 28.01.2021 | 15 | Y |  |  |
| 04.02.2021 | 22 | N | Vaccine (Pfizer-BioNTech BNT162b2) |  |
| 09.02.2021 | 27 | Y |  |  |
| 24.03.2021 | 70 | Y |  | Alpha |
| 20.04.2021 | 97 | Y |  |  |
| 17.05.2021 | 124 | Y |  |  |
| 14.07.2021 | 182 | Y |  | Delta |
| 22.07.2021 | 190 | Y | Positive SARS-CoV-2 test |  |
| 29.07.2021 | 197 | Y |  |  |
| 06.08.2021 | 205 | Y |  |  |
| 01.10.2021 | 261 | Y |  |  |
| 03.11.2021 | 294 | Y |  |  |
| 19.11.2021 | 310 | Y |  |  |
| 29.11.2021 | 320 | Y |  |  |
| 08.12.2021 | 329 | Y |  |  |
| 30.12.2021 | 351 | Y |  |  |
| 05.01.2022 | 357 | N | Vaccine (Pfizer-BioNTech BNT162b2) | BA.1 |
| 17.01.2022 | 369 | Y |  |  |
| 04.02.2022 | 387 | Y |  | BA.2 |
| 23.02.2022 | 406 | Y |  |  |
| 07.03.2022 | 418 | N | Positive SARS-CoV-2 test |  |
| 14.03.2022 | 425 | Y |  |  |
| 18.03.2022 | 429 | Y |  |  |
| 28.03.2022 | 439 | Y |  |  |
| 08.04.2022 | 450 | Y |  |  |
| 02.05.2022 | 474 | Y |  |  |
| 25.10.2022 | 650 | Y |  | BA.5 |

## Expression and purification of recombinant proteins

Recombinant SARS-CoV-2 antigens (RBD wild-type SARS-CoV-2, or its Alpha (B.1.1.7), Beta (B.1.351), Gamma (P.1) or Omicron (B1.1.529 (also known as BA.1)) variants, aa319-591) had an N-terminal BiP secretion signal and a C-terminal C-tag (N-RBD-EPEA-C) used for purification (5). The ACE2 protein (aa.1–615)-C-terminal Twin-Strep-tag (Iba, GmbH) and the spike protein (aa.35-1227)-Ctag (ΔTM-ΔFurin-CoV-PP-Ctag)) were N-terminally tagged with a BiP secretion signal (5). These antigens were synthesized by Geneart and optimized for expression in the ExpreS2 platform as previously described (5). Briefly, Schneider-2 (ExpreS2) cells were transiently transfected using transfection reagent (ExpreS2 Insect TRx5, ExpreS2ion Biotechnologies) according to manufacturer’s protocol. The Delta (AY.1) variant (N-terminally tagged with his-tag (aa319-531)) was expressed in Expi293 cells and IMAC purified.

## Enzyme linked immunoassays

Enzyme linked immunoassays were performed as previously described (1). Briefly, microtiter 96 well plates, (Nunc Maxisorb, ThermoFisher Scientific) were coated with 2 μg/mL of antigen (spike from WT SARS-CoV-2, RBD WT SARS-CoV-2 or its Alpha (B.1.1.7), Beta (B.1.351), Gamma (P.1), Delta (AY.1) or Omicron (B1.1.529 (also known as BA.1)) variants) and incubated overnight. Plates were washed three times with PBS-T and then blocked for one hour with blocking buffer (5% non-fat dry milk in PBS-T). The bound antigen was then incubated with human serum at a final dilution of 1:125. This was followed by a 1 hour incubation with the desired detection antibody: anti-human IgG-AP (Sigma Aldrich, 1:1500), anti-human IgM-AP (Sigma Aldrich, 1:1500), anti-human IgA-AP (Sigma Aldrich, 1:1500), anti-human IgG1-HRP (ThermoFisher, 1:1000), anti-human IgG2-HRP (ThermoFisher, 1:1000), anti-human IgG3-HRP (ThermoFisher, 1:1000) and anti-human IgG4-HRP (ThermoFisher, 1:1000). For AP conjugated antibodies, an incubation with 4-Nitrophenyl phosphate disodium salt hexahydrate tablets (Sigma Aldrich) dissolved in 1 × Diethanolamine Substrate Buffer (Sigma Aldrich) was performed and color development and absorbance were measured at 405 nm. For HRP conjugated antibodies, TMB substrate was added to the plate and color development and absorbance measured at 450 nm.

Plasma pools of exposed adults against each antigen were used as positive controls and to normalize plate-to-plate variability. Antibody levels were presented as arbitrary units (AU) calculated as (OD_sample_-OD_blank_)/ (OD_positive control_ -OD_blank_). In order to define a true positive result, a cut-off value was calculated for each assay as the mean + 3 SD of OD values in sera collected in 2013 from 10 healthy Danish donors (6, 7).

For competition assays (1), blocking was performed with 5% w/v BSA (Sigma Aldrich). The bound antigen was then incubated for one hour with serum (1:25 dilution) in 1% BSA. Recombinant ACE2 was added corresponding to 90% maximal binding for the WT and each VoC (RBD wild-type SARS-CoV-2, 54 nM; Alpha (B.1.1.7), 4 nM; Beta (B.1.351), Gamma (P.1), 14 nM, Delta (AY.1), 54nM; and Omicron (B1.1.529 (also known as BA.1)) 54 nM). Bound ACE2 was detected using HRP conjugated strep-tactin (IBA, 1:10 000 dilution) and TMB substrate. On each plate, a pool of immune serum was used as a positive control and commercially bought normal human serum (Sigma) as a negative control (1). ACE2 binding without antibody served as a “normal binding” control and was used to determine percent inhibition which was calculated using the formula % inhibition = 1 − (inhibited activity)/(‘normal’ binding)] × 100, after subtraction of background. In order to define a true positive result, a cut-off value was calculated for the WT and each VoC as the mean + 3SD of inhibition values in sera collected from 10 healthy Danish donors in 2013 (6, 7) (5.7%, 4.7%, 14.9%, 7.2%, 8%, 13.7% for WT, Alpha, Beta, Gamma, Delta and Omicron variants respectively).

As previously described (1), to allow for direct comparisons between all RBDs tested, IgG levels were standardized and compared as level of antibody over background cut-off. For inhibition, data was normalized for the WT and each VoC using the formula, normalized data = (percent inhibition−min/max−min) × 100 where min is the cut-off for each variant and max is the maximum value in the dataset.

**Elecsys SARS-CoV-2 antibody assays**

Elecsys Anti-SARS-CoV-2 (Roche Diagnostics, Mannheim, Germany) assays were used to measure the total SARS-CoV-2 nucleocapsid protein antibody (IgA + IgG + IgM) and the total SARS-CoV-2 spike-RBD protein antibody (IgA + IgG + IgM) level according to manufacturer’s instructions. Both assays utilize Sandwich Electro-chemiluminescence immunoassay (ECLIA) technology. For the nucleocapsid ECLIA, recombinant nucleocapsid was used and had a negative cut-off (cut-off index (COI)), of 1.0. For the spike-RBD assay, a recombinant spike protein RBD was used with a negative cut-off of 0.8 U/mL.

**Virus Neutralization assays**

SARS-CoV-2 neutralization assays were performed as previously described (5). Briefly, SARS-CoV-2, Freiburg isolate, FR-4286 (sourced from Prof. Georg Kochs, University of Freiburg) and the Delta variant (B.1.617.2) were propagated in VeroE6 cells expressing human TMPRSS2 (VeroE6-hTMPRSS2) (sourced from Prof. Stefan Pöhlmann University of Göttingen) with a multiplicity of infection (MOI) of 0.05. Supernatant containing new virus progeny was harvested 72 h post infection and concentrated by centrifugation using 100 kDa Amicon filter units (Merck). Virus titer was determined by TCID50% assay and calculated by Reed-Muench method. Human serum were prepared in DMEM at a 3-fold serial dilution starting at 1:20. Serum was mixed with SARS-CoV-2 at a final titer of 100 TCID50/well, and incubated at 4 °C overnight. Wells with no sera and a no virus (uninfected) were included as controls. The following day virus:serum mixtures were added to 2 × 10^4^ Vero E6-hTMPRSS2 cells seeded in 96-well plates, and incubated for 72 h at 37 °C, 5% CO2, before fixing with 5% formalin (Sigma-Aldrich) and staining with crystal violet solution (Sigma-Aldrich). The plates were read using a light microscope (Leica DMi1) with a camera (Leica MC170 HD) at 4 × magnification, and the cytopathic effect (CPE) was scored. Data were fitted to a four-parameter dose-response curve to interpolate IC_50_ values (GraphPad, Prism) which were used as the plaque reduction neutralization test 50% (PRNT50%) value.

**Figure S1. Binding and inhibition of plasma against SARS-CoV-2.** RBD-specific IgG levels (WT RBD, black; Alpha, blue; Beta, green; Gamma, orange) were measured at 27 days after vaccination in subject KSID (A.). Inhibition activity of KSID plasma at 27 days was measured using an ACE2 inhibition assay against WT, Alpha, Beta and Gamma variants (B.). A schematic of sample collection, circulating variants, and events (SARS-CoV-2 positive and vaccination time points) is shown in panel C.

**Table S2 Fold Delta and Omicron IgG levels over WT**

|  |  |  |
| --- | --- | --- |
| Days Post-vaccination | Delta (Fold change) | Omicron (Fold change) |
| -29 | NA | NA |
| 4 | NA | NA |
| 12 | 2.91 | 3.34 |
| 15 | 2.49 | 2.99 |
| 27 | 1.36 | 1.92 |
| 97 | 1.46 | 2.58 |
| 124 | 1.64 | 2.63 |
| 182 | 1.54 | 1.68 |
| 190 | 1.78 | 2.70 |
| 205 | 1.48 | 1.71 |
| 261 | 1.36 | 2.05 |
| 310 | 1.36 | 2.35 |
| 320 | 1.30 | 2.92 |
| 329 | 1.54 | 2.86 |
| 351 | 1.48 | 2.96 |
| 369 | 1.41 | 1.53 |
| 387 | 1.51 | 1.69 |
| 406 | 1.52 | 1.76 |
| 425 | 1.53 | 1.82 |
| 439 | 1.54 | 1.85 |
| 450 | 1.57 | 1.82 |
| 474 | 1.61 | 1.94 |
|  |  |  |

**Table S3 Fold Delta and Omicron inhibition over WT**

|  |  |  |
| --- | --- | --- |
| Days Post-vaccination | Delta (Fold change) | Omicron (Fold change) |
| -29 | NA | NA |
| 4 | NA | NA |
| 12 | NA | NA |
| 15 | NA | NA |
| 27 | 2.04 | NA |
| 97 | 4.10 | NA |
| 124 | -0.18 | NA |
| 182 | 1.63 | NA |
| 190 | -1.47 | NA |
| 205 | 1.51 | NA |
| 261 | 1.65 | NA |
| 310 | 1.27 | NA |
| 320 | 1.30 | NA |
| 329 | 1.45 | NA |
| 351 | 2.60 | NA |
| 369 | 1.03 | 1.66 |
| 387 | 1.04 | 2.73 |
| 406 | 1.10 | 5.67 |
| 425 | 1.22 | 2.24 |
| 439 | 1.41 | 2.33 |
| 450 | 1.43 | 2.31 |
| 474 | 1.33 | 2.22 |

**Table S4. Correlation of antibody subtype and inhibition**

|  |  |  |  |  |  |  |
| --- | --- | --- | --- | --- | --- | --- |
|  | **WT** | | **Delta** | | **Omicron** | |
|  | **R Squared** | **P value** | **R Squared** | **P value** | **R Squared** | **P value** |
| IgG1 | 0.5734 | **<0.0001** | 0.7741 | **<0.0001** | 0.6175 | **<0.0001** |
| IgG2 | 0.3857 | **0.002** | 0.6328 | **<0.0001** | NA* | NA |
| IgG3 | 0.05965 | 0.2733 | 0.01304 | 0.6129 | 0.01788 | 0.553 |
| IgG4 | 0.7314 | **<0.0001** | 0.896 | **<0.0001** | 0.7149 | **<0.0001** |
| IgA | 0.000122 | 0.9612 | 0.01375 | 0.6033 | 0.1577 | 0.0672 |
| IgM | 0.03571 | 0.3996 | 0.00042 | 0.9279 | 0.00437 | 0.7701 |
| IgG | 0.8964 | **<0.0001** | 0.6764 | **<0.0001** | 0.4096 | **0.0013** |

**References**

1. Walker MR, Podlekareva D, Johnsen S, Leerhoy B, Fougeroux C, Sogaard M, et al. SARS-CoV-2 RBD-specific antibodies induced early in the pandemic by natural infection and vaccination display cross-variant binding and inhibition. Viruses. 2022;14(9).

2. Johnsen S, Sattler SM, Miskowiak KW, Kunalan K, Victor A, Pedersen L, et al. Descriptive analysis of long COVID sequelae identified in a multidisciplinary clinic serving hospitalised and non-hospitalised patients. ERJ Open Res. 2021;7(3):205-17.

3. Khare S, Gurry C, Freitas L, Schultz MB, Bach G, Diallo A, et al. GISAID's role in pandemic response. China CDC Wkly. 2021;3(49):1049-51.

4. Ritchie H, Mathieu E, Rodés-Guirao L, Appel C, Giattino C, Ortiz-Ospina E, et al. Coronavirus Pandemic (COVID-19) OurWorldInData.org. Retrieved from: '<https://ourworldindata.org/coronavirus'2020> [

5. Fougeroux C, Goksoyr L, Idorn M, Soroka V, Myeni SK, Dagil R, et al. Capsid-like particles decorated with the SARS-CoV-2 receptor-binding domain elicit strong virus neutralization activity. Nat Commun. 2021;12(1):324.

6. Walker MR, Knudsen AS, Partey FD, Bassi MR, Frank AM, Castberg FC, et al. Acquisition and decay of IgM and IgG responses to merozoite antigens after Plasmodium falciparum malaria in Ghanaian children. PLoS One. 2020;15(12):e0243943.

7. Partey FD, Castberg FC, Sarbah EW, Silk SE, Awandare GA, Draper SJ, et al. Kinetics of antibody responses to PfRH5-complex antigens in Ghanaian children with Plasmodium falciparum malaria. PLoS One. 2018;13(6):e0198371.
